# Linguistic aspects of auditory verbal hallucinations in schizophrenia: sentence length and cohesion

**DOI:** 10.1192/j.eurpsy.2026.12178

**Published:** 2026-07-31

**Authors:** P. del Olmo-Encabo, P. Fuentes-Claramonte, J. Soler-Vidal, F. Neuhaus, L. López-Araquistain, L. Barbosa, P. Salgado-Pineda, S. Sarró, J. Rosselló-Ximenes, P. J. McKenna, E. Pomarol-Clotet

**Affiliations:** 1 FIDMAG Germanes Hospitalàries Research Foundation, Sant Boi de Llobregat; 2 ISCIII, Cibersam, Barcelona, Spain; 3 Maastricht University, Maastricht, Netherlands; 4 Hospital Sant Rafael; 5 Departament de Filologia Catalana i Lingüística General, Universitat de Barcelona, Barcelona, Spain

## Abstract

**Introduction:**

Auditory verbal hallucinations (AVH) are a core clinical feature of schizophrenia that by definition involve language. It has been commented that the voices of patients with schizophrenia tend to speak in short sentences, but this has never been examined empirically. Prior studies have found that the speech of patients with schizophrenia shows a linguistic abnormality, reduced numbers of cohesive ties – the devices that link individual sentences into texts – compared to healthy subjects (Rochester and Martin, 1979). However, it is not known whether changes in cohesive tie numbers also characterize hallucinated schizophrenic speech.

**Objectives:**

To compare sentence length and cohesive tie numbers in speech samples from healthy controls , and verbatim transcripts of hallucinated speech from a group of schizophrenia patients with very frequent auditory hallucinations.

**Methods:**

Speech samples (HC, N = 20) and verbatim transcripts of hallucinations (AVH patients, N=16) were segmented into sentences. The groups were matched by sex and age. Sentence length was calculated as the number of words per sentence. Cohesion analysis was carried out using the taxonomy in Halliday & Hasan (1976), identifying examples of four of their five types: reference, conjunction, ellipsis, lexical cohesion (there were too few instances of a fifth type, substitution for analysis).

**Results:**

After covarying for sex, age, premorbid and current IQ and frequency of incomprehensible fragments, sentence length was significantly shorter in hallucinated speech than healthy real speech (t_(48)_ = 4.25, p < 0.001) . Transcripts of AVH speech showed reduced cohesion across all ties examined (all t(34) > 3.68 , all p > 0.01) except lexical cohesion (t(48) = 1.62, p = 0.25).

**Conclusions:**

This study finds preliminary support for the view that hallucinated voices in schizophrenia speak in short sentences. AVH speech also exhibits cohesion breakdown, similarly to the (real) speech of patients with schizophrenia. Both findings require further confirmation, e.g., by comparing hallucinated speech with real speech in the same patients.

**Disclosure of Interest:**

None Declared

